# Temporal trends in vena cava filter implantation in public health system inpatients: an 11-year analysis of the largest city in Brazil

**DOI:** 10.1590/1677-5449.20210186

**Published:** 2022-04-05

**Authors:** Dafne Braga Diamante Leiderman, Marcelo Fiorelli, Marcelo Passos Teivelis, Nickolas Stabellini, Edson Amaro, Nelson Wolosker

**Affiliations:** 1 Hospital Israelita Albert Einstein – HIAE, São Paulo, SP, Brasil.; 2 Universidade de São Paulo – USP, Faculdade de Medicina, Hospital de Clínicas, São Paulo, SP, Brasil.; 3 Faculdade Israelita de Ciências Médicas Albert Einstein – FICMAE, São Paulo, SP, Brasil.; 4 Universidade de São Paulo – USP, São Paulo, SP, Brasil.

**Keywords:** inferior vena cava filter, epidemiology, public health system, mortality rate, thrombosis, pulmonary embolism, filtro de veia cava, epidemiologia, sistema público de saúde, taxa de mortalidade, trombose, embolia pulmonar

## Abstract

**Background:**

Vena cava filter implantation is considered a simple procedure, which can lead to overuse and over-indication. It is nevertheless associated with short and long-term complications.

**Objectives:**

The goals of this study were to evaluate rates of vena cava filter implantation conducted by Brazil’s Unified Public Health System, analyzing in-hospital mortality and migration of patients from other cities seeking medical attention in São Paulo.

**Methods:**

This study analyzed all vena cava filter procedures conducted from 2008 to 2018 in the city of São Paulo and registered on the public database using a big data system to conduct web scraping of publicly available databases.

**Results:**

A total of 1324 vena cava filter implantations were analyzed. 60.5% of the patients were female; 61.7% were under 65 years old; 34.07% had registered addresses in other cities or states; and there was a 7.4% in-hospital mortality rate.

**Conclusions:**

We observed an increase in the rates of use of vena cava filters up to 2010 and a decrease in rates from that year onwards, which coincides with the year that the Food and Drug Administration published a recommendation to better evaluate vena cava filter indications.

## Resumo

**Contexto:**

O implante de filtro de veia cava é considerado um procedimento de baixa complexidade, o que pode resultar em indicação excessiva. No entanto, não é isento de complicações a curto e longo prazo.

**Objetivos:**

Avaliar as taxas de implantes de filtro de veia cava realizados pelo Sistema Único de Saúde e a origem geográfica e mortalidade intra-hospitalar dos pacientes.

**Métodos:**

Foi conduzida uma análise em um banco de dados públicos referente às taxas de implantes de filtro de veia cava realizados de 2008 a 2018 na cidade de São Paulo, utilizando o sistema de *big data*.

**Resultados:**

Foram analisados 1.324 implantes de filtro de veia cava financiados pelo Sistema Único de Saúde. Identificou-se tendência de aumento da taxa de implantação até 2010 e de redução dos números após esse período. Do total de pacientes, 60,5% eram do sexo feminino; 61,75% tinham menos de 65 anos; e 34,07% possuíam endereço oficial em outra cidade ou estado. A taxa de mortalidade intra-hospitalar foi de 7,4%.

**Conclusões:**

Observamos aumento das taxas de implante de filtro de veia cava até 2010 e redução das taxas após esse período, o que coincide com o ano em que a organização norte-americana Food and Drug Administration publicou uma recomendação para melhor avaliar as indicações de filtros.

## INTRODUCTION

Venous thromboembolism (VTE), manifesting as deep vein thrombosis (DVT) or pulmonary embolism (PE), is a serious and potentially fatal disease affecting up to 5% of the general population.^1^ The treatment of choice for VTE is full anticoagulation;^2^^-^5 however, some patients are contraindicated for anticoagulation or have recurrent VTE even with adequate anticoagulation^6^ and require implantation of a vena cava filter (VCF) to prevent pulmonary embolism (PE) mechanically.^7^ The purpose of vena cava filters (VCFs) is to decrease recurrent PE or PE-related mortality.

Since it is a percutaneous procedure, VCF implantation is considered a simple procedure, which can induce overuse and over-indication of the treatment. VCF implantation is associated with short and long-term complications^8^^,^^9^ and its benefits for preventing PE and reducing mortality rates have been assessed by recent studies.^10^

In response to studies about VCF complications, the United States (US) Food and Drug Administration (FDA) published a recommendation in 2010 that VCF indications should be evaluated better and filter retrieval rates should be increased. Since that event, the international published literature reported a reduction in VCF implantation rates in the US and Europe,^10^ but there have been no Brazilian or Latin American epidemiologic studies showing the impact of these publications on trends related to VCF placement in Brazil.

The public healthcare system in Brazil is called the Sistema Único de Saúde (SUS) and is a tax-financed, universal, equitable, and integral government-run system.^11^ Everyone has access to it, but in practice the SUS provides care to 75% of the population. The remaining 25% make use of the supplementary private health system, in which costs are covered individually either by the user or their employer.^12^ The public healthcare system provides treatments for all diseases, but there are long waiting lines most of the time and simpler hospitality than in hospitals in the supplementary private health system. The SUS stores some information about surgeries on a public database, which is anonymized and is not a medical record, but includes all public hospitals in each town in which VCF are implanted and paid for by the SUS.

Brazil has 5570 towns. São Paulo is the largest city and has the second highest Human Development Index (HDI) in Brazil (0.783).^13^ In 2016, it was estimated that the city’s population exceeded 12 million people, 5 million of whom were uniquely dependent on the public health system, a proportion that is larger than some states or even countries. Furthermore, São Paulo is the most important state capital economically and has greater availability of the most modern treatments with the latest technology in terms of equipment and drugs. It therefore centralizes a large number of patients from other cities and states seeking health services and solutions for serious diseases.

Notable gains have been achieved in global health over the past 25 years, but progress in health has not been uniform across countries. It is important to know the real-world results in large populations of entire cities the size of São Paulo, which has a population as large as some countries. Additionally, it is essential to understand the trends of some popular surgical procedures across countries.

The goals of this study were to evaluate VCF implantation rates performed within the public health system in the largest city in Brazil between 2008 and 2018 using a big data system and to evaluate in-hospital mortality and migration of patients from other cities seeking medical care in São Paulo.

## METHODS

Data were retrieved from the TabNet platform, a public health information application developed by DATASUS, the Health Informatics Department of the Brazilian Ministry of Health.^14^ The TabNet system provides open data regarding procedures performed within the Brazilian public health system by hospitals adequately accredited as vascular surgery centers. Such accreditation is a prerequisite for hospitals to receive remittances from the public health system relative to the procedures they perform.

The TabNet platform allows 22 possible search selections for rows, 16 for columns, and 8 for content, resulting in 2816 formatting possibilities for searches that are then subdivided by monthly periods.

The institutional Ethics Committee approved this study (CAAE 35826320.2.0000.0071; Decision 4.321.508). All data provided by DATASUS and TabNet are anonymized. For this reason, the Institutional Review Council (Conselho de Revisão Institucional, IRB) waived the requirement for informed consent forms.

Statistics referring to vascular surgery procedures for VCF implantation were selected for the period 2008 to 2018 on the TabNet platform maintained by the Municipal Health Secretariat of São Paulo, Brazil. The data selected and analyzed included sex, age, municipality of residence, number of procedures performed, and in-hospital mortality.

Only one procedure was evaluated for VCF implantation, according to codes established by the Brazilian public healthcare system for management of procedures and medications - SIGTAP (Sistema de Gerenciamento da Tabela de Procedimentos, Medicamentos e OPM). The procedure code selected for analysis was vena cava filter implantation (04.06.04.014-1).

All data were collected from public access sites through computer programs for automated content access (web scraping). These automated navigation codes were programmed in Python language (v. 2.7.13, Beaverton – Oregon – USA) using the Windows 10 Single Language operating system.

The data collection, platform field selection, and table adjustment steps were performed using the selenium-webdriver packages (v. 3.1.8, Selenium HQ, several collaborators worldwide) and pandas (v. 2.7.13, Lambda Foundry, Inc. and PyData Development Team, New York, USA).

The automated navigation code (web scraping) presents a central structure with 14 adjustable search phases according to the different search filters available within the platform. The Mozilla Firefox browser (v. 59.0.2, Mountain – California – USA) and geckodriver webdriver (v 0.18.0, Mozilla Corporation, Bournemouth, England) were used.

Following collection and treatment, all data were organized and grouped in a spread sheet using Microsoft Office Excel 2016® (v. 16.0.4456.1003, Redmond – Washington – USA) software. The table was formatted into the following columns: total number of patients operated and mortality (absolute and percentage).

We used Poison Regression to analyze the number of procedures over time. Statistical analyses were performed using IBM-SPSS for Windows version 20.0, and tests were performed with a significance level of 5%.

## RESULTS

A total of 1324 VCF implantations paid for by the SUS were performed in São Paulo city from 2008 to 2018. Most patients were female (60.5%). Patients distribution by age was as follows: 817 procedures (61.7%) in patients under 65 years old and 507 procedures (38.3%) in patients over 65 years old. In 65.93% of the procedures, patients had registered residential addresses in São Paulo, whereas 34.07% had registered addresses in other cities or states.

**Figure 1:**
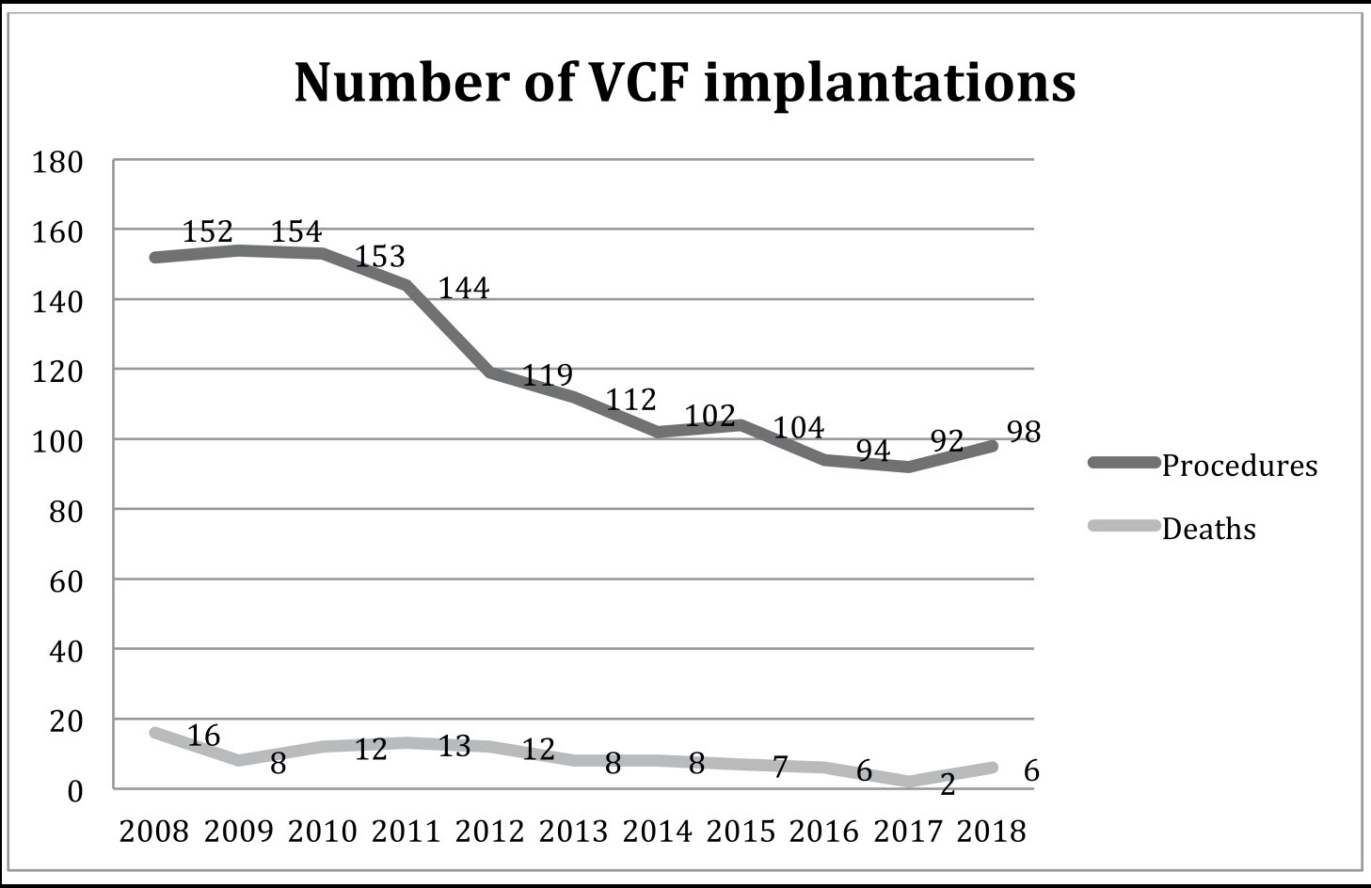
Absolute number of VCF implantations per year from 2008 to 2018.

The number of procedures per study year is presented in Figure 1.

The number of procedures per year has decreased since 2010 (p<0.001). There were 98 in-hospital deaths observed over the years evaluated, equating to 7.4% of the patient sample.

## DISCUSSION

This study presents publicly available data from the DATASUS database.^14^ DATASUS is the Health Informatics Department of the Brazilian Ministry of Health, created in 1991. The SIGTAP system mentioned previously is the Brazilian public health service’s management system for procedures and medications. This platform provides reliable information regarding institutions within the public health system that are accredited to perform specific procedures and it also constitutes an invaluable tool to aid certain aspects of financial decision-making related to public healthcare.

An important limitation to our study is that only information about accredited institutions is included in the database, possibly excluding procedures performed in institutions not listed within the DATASUS databases or any that omitted this code in the surgery description. We acknowledge that, as with any population in code-based research, there may have been some miscoding and/or loss of data. However, there were probably few such procedures excluded, considering that VCF implantation has only one option for a corresponding procedure code and the surgeon has to justify the use of the filter, but there may have been some loss of information on surgeries where VCF implantation was not the main procedure. Omission of the code for the VCF implantation procedure results in nonpayment of these items by the SUS.

Additionally, although the total number of procedures was analyzed, this database is not a medical record database and it was not possible to determine the VCF indications, complications, reoperation rates, or related clinical information because it is an anonymized database. On the other hand, this is an original analysis of a large study population that was made possible by using big data information.

Despite the public nature of the data analyzed in this study, manually driven collection of information, however technically viable, would demand a significant time investment. To facilitate and expedite the data collection process, an automated navigation code programming tool was used. This was shown to be a faster and simpler method of collection of the publicly available data, enabling analysis of a significantly larger number of procedures over a longer period of time.

In our study, the demographic data were similar to data in literature published previously.^15^ Other authors have observed a slight predominance of female patients, ranging from 50.1% to 60.4%,^15^^-^17 and in common with this, 60.5% of the patients treated in the present sample were female. In previously published studies, the mean age was 58.1-67 years. In our study, 61.7% of patients were under 65 years old. This information is expressed in age ranges in our database, so it was not possible to calculate the mean age.

The preponderance of female individuals is due to a higher prevalence of VTE in females in the general population, and some risk factors include the use of hormonal drugs, presence of varicose veins, gestation, and long postoperative surgeries.^18^ Additionally, active cancer is found in approximately 20% of patients diagnosed with venous thromboembolism (VTE).^7^^,^^19^^,^^20^ Additionally, VTE is more prevalent in patients with advanced and metastatic cancer, and has a high prevalence in breast and gynecological cancer patients.^6^^,^^19^^,^^21^^-^23 Most of our data are from tertiary hospitals and large oncological hospitals, which explains the age and sex prevalence findings and may be associated with the 7.4% in-hospital mortality rate in our sample. VTE is the second largest cause of death among patients with active neoplasms,^24^^-^26 and a large proportion of these patients have high risk or active bleeding and require VCF implantation.^27^ Additionally, patients in the ICU or with long hospital stays have a higher rate of VTE and higher mortality. Therefore, this mortality rate is not primarily associated with immediate complications of VCF implantation procedures, but illustrates the seriousness of these patients’ health status.^27^

Since all information was sourced from a secondary database (DATASUS), all data analyzed were anonymized. Therefore, it was not possible to determine the cause of death or medical history of deceased patients or identify direct correlations between death and the surgical procedure. Detailed statistics or complications regarding mortality associated with VCF implantation were not the aim of this study, and the international literature includes good studies regarding this topic.^8^^,^^28^^,^^29^

VCF implantation became a well-known and widespread procedure at the end of the 1990s, and American and European studies showed a notable increase in the number of procedures up until 2010. Rates of inferior VCF implantation range from 12% to 17% in all patients with VTE.^10^^,^^31^^,^^32^ Previous studies have shown that the US has the highest rates of VCF implantation,^33^ and the rate had exponentially increased in the US over 2 decades, growing at a rate of 5.81% from 2005 to 2010.^34^^,^^35^ Our data start in 2008 and rates also increased up until 2010. In 2005, the PREPIC randomized study increased concerns regarding the true benefit of VCF implantation. This study concluded that inferior VCF decreased rates of recurrent PE with no effect on short or long-term mortality, but was associated with significantly higher rates of recurrent deep venous thrombosis (DVT).^10^ After that, other studies questioned the benefits, the increase in complication rates, and the low rates of VCF retrieval^8^^,^^9^^,^^36^ and, in 2010, the FDA issued a safety advisory to physicians about the dangers associated with prolonged filter implantation, sparking controversy about whether the risks outweighed the benefits of VCF placement. As an unsurprising response to this warning, VCF usage declined by as much as 6.48% for VCF placement in 2014 in the US.^37^ With the litigious environment developing around VCF in the post FDA-advisory era and with all eyes open with regard to the subject, this international scenario apparently led to a decrease in VCF implantation in Brazil, as was shown in our study.^38^ Our data showed that the FDA warning might have affected Brazilian rates, with a 40% decrease in VCF implantation from 2010 to 2018.

Another factor that may be associated with this decrease in VCF implantation is improvements of hemostatic techniques that are now solving some of the cases that had been previously been contraindicated for anticoagulation.^36^ Additionally, doctors are trying to better define the indications for VCF implantation. An American study reported that doctors agreed that appropriate indications for VCF use were present in only 51% of cases of VCF placement.^32^

In addition, the literature reported an increase in retrieval rates after 2010, probably in response to that warning. Our study could not show this effect in Brazil because we did not have a code specifically for VCF retrieval in our procedure code list. We did have a code to describe the removal of any intravascular foreign material, which could be a catheter, a type of endovascular material such as a guidewire or embolization coil, or a filter.

Regarding the primary residency address, most patients were living in São Paulo, but 34.07% had a residency address in another city or state. This finding contrasts with that of more complex diseases demanding a referral center for treatment, as in the case of carotid stenosis: 36.3% of patients who underwent surgical treatment for cerebrovascular disease in São Paulo over a 10-year period were resident elsewhere.^39^ VTE is a frequent complication of serious diseases, and a large percentage of patients require VCF implantation before surgery or have other contraindications for anticoagulation. Since São Paulo is a center of healthcare excellence, people spontaneously travel to this capital seeking complex medical care.

## CONCLUSION

In a city whose population surpasses those of some European countries, VCF implantation procedures paid for by the public healthcare system followed the worldwide trend, with rates increasing up until 2010 and decreasing from that date onwards. A total of 1324 VCF implantations were performed in São Paulo city, there was a 7.4% rate of in-hospital death, and 34.07% of patients had registered addresses in other cities or states.
